# Is the onset of disabling chronic conditions in later childhood associated with exposure to social disadvantage in earlier childhood? a prospective cohort study using the ONS Longitudinal Study for England and Wales

**DOI:** 10.1186/1471-2431-13-101

**Published:** 2013-06-26

**Authors:** Clare M Blackburn, Nicholas J Spencer, Janet M Read

**Affiliations:** 1 Warwick Medical School, University of Warwick, Coventry CV4 7AL, UK

**Keywords:** Children, Chronic Conditions, Disabilities, Disadvantage, Limiting Long-term Illness, Socio-economic Factors

## Abstract

**Background:**

The aetiology of disabling chronic conditions in childhood in high income countries is not fully understood, particularly the association with socio-economic status (SES). Very few studies have used longitudinal datasets to examine whether exposure to social disadvantage in early childhood increases the risk of developing chronic conditions in later childhood. Here we examine this association, and its temporal ordering, with onset of all-cause disabling chronic later childhood in children reported as free from disability in early childhood.

**Methods:**

The study comprised a prospective cohort study, using data from the Office for National Statistics Longitudinal Study (ONSLS) for England and Wales. The study sample included 52,839 children with complete data born between 1981–1991 with no disabling chronic condition/s in 1991. Index cases were children with disability recorded in 2001. Comparison cases were children with no recorded disability in 1991. A socio-economic disadvantage index (SDI) was constructed from data on social class, housing tenure and car/van access. Associations were explored with logistic regression modelling controlling sequentially for potentially confounding factors; age, gender, ethnicity and lone parenthood.

**Results:**

By 2001, 2049 (4%) had at least one disability. Socio-economic disadvantage, age, gender and lone parenthood but not ethnicity were significantly associated with onset of disabling chronic conditions. The SDI showed a finely graded association with onset of disabling chronic conditions in the index group (most disadvantaged OR 2·11 [CI 1·76 to 2·53]; disadvantaged in two domains OR 1·45 [CI 1·20 to 1·75]; disadvantaged in one domain OR 1·14 [CI 0·93 to 1·39] that was unaffected by age, gender and ethnicity and slightly attenuated by lone parenthood.

**Conclusion:**

To our knowledge, this is the first study to identify socio-economic disadvantage in earlier childhood as a predisposing factor for onset of all-cause disabling chronic conditions in later childhood. Temporal ordering and gradation of the response indicate socio-economic disadvantage may play a causal role. This suggests that targeting preventative efforts to reduce socio-economic disadvantage in early childhood is likely to be an important public health strategy to decease health inequalities in later childhood and early adulthood.

## Background

Disabling chronic conditions in childhood continue to be a significant global public health issue, [1] with an estimated 200 million children age 0–17 experiencing some kind of disability [2]. While the majority of these children live in low and middle income countries, such conditions remain a significant issue for high income countries, where prevalence estimates range between 1.5 to 10 percent [3] and are increasing in some countries [4]. The aetiology of disabling chronic conditions in childhood in high income countries is not fully understood, particularly the association with socio-economic status (SES). Many cross-sectional studies have documented the association of low SES with disabling chronic conditions, [5-8] but studies of this design cannot answer questions about causality or temporal sequencing [9]. As highlighted by the World Report on Disability, even in developed countries longitudinal studies to establish causal relations between low SES and disability are rare [1]. Studies that have used longitudinal designs to explore pathways from early childhood socio-economic disadvantage to disabling chronic conditions and poor health in later childhood (see for example [10,11] have often failed to report on whether the child already had an existing disabling chronic condition in early childhood and, thus, the temporal direction cannot be shown from their findings.

Although many children with disabling chronic conditions are able to lead rich and fulfilling lives, in addition to low SES, they are more likely than other children to experience social exclusion, [1,12] have comorbidities and poorer adult health [13,14] and have other family members who also experience health issues and disability [15]. As a result, the impact on children, families and health, social care and education systems is substantial [16]. Reducing both the prevalence of childhood disabling chronic conditions and their impact on the lives of children and their families is important, but requires robust evidence on their causes to enable development of effective policy and interventions.

The aim of this study was to examine the relationship between socio-economic disadvantage and the onset of childhood disabling conditions within a prospective cohort study in England and Wales. Using data from the UK Office of National Statistics Longitudinal Study (ONSLS) we report on children who were identified as being free from disabling chronic conditions in early childhood (0–10 years) and examine whether exposure to socio-economic disadvantage during this period increased their risk of developing a disabling chronic condition in later childhood and early adulthood (10–20 years). To our knowledge, this is the first study to examine this association and its temporal ordering for all-cause disabling chronic conditions in childhood.

## Methods

### Study design

With the permission of the Office for National Statistics to access and use ONSLS data, we conducted a cohort study, with a 10 year follow-up period. The ONSLS is a representative 1% sample of individuals in England and Wales, initiated at the time of the 1971 Population Census [17]. Study members were children born on one of four selected dates of birth in 1971 or subsequent census years. Individuals were tracked through subsequent censuses (1981, 1991 and 2001) and their data linked. The ONSLS has a large sample of children and thus has sufficient power to account for the potential confounding of the relationship between socio-economic disadvantage and disabling chronic conditions. In addition to data on cohort members, the ONSLS also contains data on individuals (non-members) identified at each census as living in the same households as cohort members. Thus data on the socio-economic circumstances of the households within which the study children live are available. Data files were created, containing only the 1991 and 2001 census data fields relevant to our study, on children born between 1981 and 1991 who became ONSLS members and who have been tracked in the 1991 and 2001 censuses.

### Index and comparison groups

Children with disabling chronic conditions were identified from a question asked in the 1991 and 2001 Population Census of the household reference person. Children for whom the household reference person responded positively to the following question were classified as having a disabling chronic condition:

‘Do you have any long-term illness, health problem, or handicap which limits your daily activities or the work you can do?’

In this cohort study, all children with no disability at 1991 census were included, and these were classified on the basis of their 2001 data. The index group of interest was all ONSLS child members born between 1981 and 1991 reported to have a disabling chronic condition in 2001 but not in 1991. The comparison group comprised ONSLS child members born between 1981 and 1991, who were reported as not having a disabling chronic condition in either 1991 or 2001. Data were extracted on both the index and comparison groups.

### Independent variables of interest

The independent variables of interest, extracted from the 1991 member and non-member data files, were as follows:

Highest household social class: data were extracted from the non-members’ data file collapsed into 6 categories: I - Professional; II- Managerial & Technical; IIINM - Skilled non-manual; IIIM – Skilled manual; IV - Partly skilled; V– Unskilled. Armed Forces households and inadequately described social class were treated as missing data.

Housing tenure: data extracted from the members’ data file collapsed into 2 categories: Owner-occupied and Rented/other accommodation. Those in communal establishments were treated as missing data as no data were available on social class of household members.

Car/van ownership: data extracted from the members’ data file in 4 categories: no car/van; one car/van; two cars/vans; three or more cars/vans).

As no standardised deprivation score was available in the dataset, these three variables were used to construct a household socio-economic disadvantage aggregate index (SDI) that combined the available individual measures of socio-economic exposures into a continuous measure and allowed the gradation of onset by socio-economic disadvantage to be examined. Each of the available variables was dichotomised (housing tenure: owner occupied v rented/other; social class of household: high (1–4) v low (5–6); Car/van ownership: 1 or more cars/vans v 0 cars/vans) and summed to generate a socio-economic disadvantage aggregate index. Each variable was scored as follows: 0 indicates no disadvantage, score of 1 indicates disadvantage. Scores were summed across variables to give an aggregate score of 0–3, where 0 = not disadvantaged on any and 3 = disadvantaged on all 3 factors.

### Confounding variables

A number of factors such as age, sex, ethnicity and family status have been shown in other studies to be associated with childhood disability and can confound the relationship between childhood disability and socio-economic disadvantage. For this study, available potential confounding variables were: child’s age (in years in 1991 extracted from the members’ data file); child’s sex (extracted from the members’ data file); child’s ethnicity extracted from the members’ data file and collapsed into 5 categories: White UK/European; Black British/Caribbean/African; Indian; Pakistani and Bangladeshi; Other); family status (in 1991 extracted from the members’ data file and collapsed into 2 categories: lone parent with children; married/co-habiting pair with children, with all other categories treated as missing data).

### Data analysis

#### Missing data

Children with missing data were omitted from the analysis. Cross tabulations were run to establish any differences between children with complete data and those with missing data using the Pearson Chi-square test.

#### Main analyses

In order to examine the associations between individual, family and socio-economic factors and disabling chronic conditions, the Pearson Chi-square test and Chi-square for linear trend were used. At the multivariable level, we explored the associations between socio-economic disadvantage in early childhood and developing disabling chronic conditions up to 10 years later using multiple logistic regression modelling, introducing sequentially potential confounders of the association (age, sex, ethnicity and family status). Logistic regression models were fitted on the index and comparison groups to produce odds ratios with 95% confidence intervals. First, the SDI variable alone was entered. Second, the SDI was entered with age and sex. Third, ethnicity (white vs. all other) was added along with age and sex. At the final stage, family status was added together with age, sex and ethnicity. Only those children with complete data for all variables were included in the regression models. Statistical significance was set at the 5% level (P ≤0.05). All analyses were carried out in SPSS V16 and undertaken on secure computers at the ONS Virtual Microdata Laboratory, London.

## Results

Data were available on 61603 children aged 0–10 years who became LS members between 1981 and 1991. Of these, 53852 children had complete data: data for the main independent variable of interest, the SDI, were missing for 7751 children. Data on lone parenthood were missing for 766 children; however, all also had missing SDI data. There were no missing data for age, gender or ethnicity. Comparing housing tenure and car ownership of those with missing data against those with complete data indicated that children with missing data tended to live in more disadvantaged households (rented/other: missing (81·4%) v. complete (24·3%); no car ownership: missing (49·8%) v. complete (17·2%). Of the 53852 children with complete data, 1013 children either had a disabling chronic condition in 1991 only, or in both 1991 and 2001, and were excluded from analyses. The final cohort comprised 52839 children, with 2049 and 50790 in the index and comparison groups respectively. Table 1 shows their socio-demographic characteristics in 1991.

**Table 1:** Socio-demographic characteristics of the sample, 1991

**Characteristics**	**Index group**	**Comparison group**	**OR (95% CI) and p value**
	**(no disabling chronic condition 1991 but onset of disabling chronic condition by 2001)**	**(no disabling chronic condition 1991 or 2001)**	
	**n = 2049**	**n = 50790**	
*Sex:*			
Boys	1131 (55·2%)	25794 (50·8%)	1.20 (1.10,1.31)
Girls	918 (44·8%)	24996 (49·2%)	p<0·001
*Mean age (2001)*	14·7	14·56	1.02 (1.01,1.03)
			p<0·01
*Ethnicity:*			Compared with White:
White	1878 (91·7%)	46926 (92·4%)	0.98 (0.91,1.07)
Black	117 (5.7%)	2616 (5·2%)	p<0.1917
Indian	11 (0·5%)	241 (0·5%)	
Pakistani/Bangladeshi	27 (1·3%)	769 (1·5%)	
Other	16 (0·8%)	236 (0·5%)	

*Family status in 2001:*			
Lone parent household	328 (16·0%)	5718 (11·3%)	1.55 (1.41,1.71)
			p<0·001
*Socio-economic Disadvantage Index in 1991*:			
Disadvantaged in 3	169 (8·2%)	2337 (4·6%)	2·20(1·86,2·59)
Disadvantaged in 2	258 (12·6%)	4114 (8·1%)	1·49(1·25,1·79)
Disadvantaged in 1	504 (24·6%)	10405 (20·5%)	1·15(0·94,1·41)
Not disadvantaged	1118 (54·6%)	33934 (66·8%)	1(reference)
			p<0·001 (linear trend)

In bivariate analysis, boys (OR 1.20 [1.10,1.31]) older children (OR 1.02 [1.01,1.03]), lone parent household (OR 1.55 [1.41,1.71]), no car ownership (OR1.38 [1.30,1.45]), living in rented accommodation (OR 1.79 [1.65,1.93]), lower social class (OR 1.74 [1.28,2.36]) and higher levels of socio-economic disadvantage in 1991 (2.20 [1.86,2.59]) were all associated with significantly increased odds of developing disabling chronic conditions between 1991 and 2001 (Table 1). Social gradients were shown in car ownership, social class and the SDI. Ethnicity (white vs. all other) was not significantly associated with the development of disabling chronic conditions in later childhood (OR 0.98 [0.91,1.07]).

In the logistic regression models (Table 2), the odds associated with increasing socio-economic disadvantage were unchanged by the addition of age and gender and ethnicity (models 2 & 3 in Table 2) and only slightly attenuated by the addition of lone parenthood (model 4 in Table 2). For children in the most socio-economically disadvantaged households in 1991, the odds of developing disabling chronic conditions by 2001 were more than twice those in the least disadvantaged households (Table 2). The odds were graded by level of socio-economic disadvantage and, although the odds ratio for those disadvantaged in one domain only was not statistically significant, the odds ratio was greater than one and consistent with a linear trend with disadvantage. The significantly increased odds of older children and boys developing disabling chronic conditions by 2001 remained unchanged. Living in a lone parent household in 1991 was no longer significantly associated with developing disabling chronic conditions by 2001. The population attributable risk for the onset of disabling chronic conditions in 2001 by socio-economic disadvantage in 1991 was 17.5%: if all children had the same chances as those reported as not exposed to socio-economic disadvantage in earlier childhood, there would have been 359 fewer children reported as having disabling chronic conditions in later childhood.

**Table 2:** Odds of a child/young person without any reported disabling chronic condition in 1991 being reported as having developed a disabling chronic condition by 2001

**Independent variables**	**Model 1**	**Model 2**	**Model 3**	**Model 4**
	***OR (95% CI)***	***OR (95% CI)***	***OR (95% CI)***	***OR (95% CI)***
Socio-economic disadvantage index				
No disadvantage	Reference	Reference	Reference	Reference
Disadvantaged in 3	2·20 (1·86,2·59)	2·21 (1·87,2·62)	2·20 (1·87,2·62)	2·11 (1·76,2·53)
Disadvantaged in 2	1·49 (1·25,1·79)	1·50 (1·26,1·80)	1·51 (1·26,1·81)	1·45 (1·20,1·75)
Disadvantaged in 1	1·15 (0·94,1·41)	1·15 (0·94,1·41)	1·16 (0·95,1·41)	1·14 (0·93,1·39)
Sex (girls as reference)	-	1·20 (1·10,1·31)	1·20 (1·10,1·31)	1·20 (1·10,1·31)
Age (<1 as reference)	-	1·02 (1·01,1·03)	1·02 (1·01,1·03)	1·02 (1·01,1·03)
Ethnicity* (White as reference)			0·99 (0·92,1·08)	0·99 (0·92,1·08)
Family status (2 adult household as reference)	-	-	-	1·11 (0·97,1·27)

*Ethnicity – white vs. all other.

## Discussion

To our knowledge this is the first paper to examine, in a longitudinal dataset, the association between socio-economic disadvantage in early childhood and onset of all-cause disabling chronic conditions in later childhood, where the temporal ordering is clear. Our analysis of a representative sample of children in England and Wales, showed that children who lived in socio-economically disadvantaged households when they were aged 0–10 years and who were reported as not having any disabling chronic condition at this time point were at greater risk of developing disabling chronic conditions in later childhood (when they were 10–20 years) than children who lived in more advantaged households in earlier childhood. This risk was finely graded by the extent of disadvantage, with the odds of developing a disabling chronic condition increasing significantly as the level of household socio-economic disadvantage rose. The odds ratio for disadvantage in one domain only failed to reach statistical significance at the 5% level but was above unity consistent with a linear trend. The grading suggests there may be a ‘dose-response’ relationship of socio-economic disadvantage in early childhood with disabling chronic conditions in later childhood. Our findings on disabling chronic conditions are consistent with the evidence base on socio-economic disadvantage as a determinant of broader child health. This association has been reported in high income countries with different child healthcare systems, including the UK; [18,19] Canada; [18,20] the USA; [11,16], and in the Nordic countries; [21,22] and among children across all age groups [19].

We identified only three studies using longitudinal data to report on the role of income and SES in the later onset of disabling chronic conditions. Two [23,24] of these included children with a range of conditions, not all of which would be classified as chronic, activity limiting or disabling and the third reported on the association of various SES indicators on onset of specific mental health disorders but not on all disabling chronic conditions [25].

Although some studies have found a link between lone parenthood and child health [26,27] our study did not, but was consistent with other reports that showed the effect of lone parenthood on child health outcomes was accounted for by socio-economic disadvantage [28,29]. In our study, ethnicity showed no statistically significant association with onset of disabling chronic conditions, in contrast to studies in other high income countries which have documented persisting ethnicity effects even after control for SES [30].

### Strengths and limitations of the study

The main strength of our study is the utilisation of a longitudinal dataset, based on Population Census data, with a representative 1% sample of individuals. This generated a large sample of children with data at two time points in a study with sufficient power to account for the potential confounding of the relationship of onset of disabling chronic conditions with socio-economic disadvantage by factors such as age, sex, lone parenthood and ethnicity. The paucity of longitudinal datasets in the UK and elsewhere, with data on disabling chronic conditions and sufficient sample sizes to look examine associations and temporal ordering, make this an important data source. The study has a number of limitations which should be taken into account when interpreting the findings. Participation in the UK census is high (the overall participation rate in 2001 was 94%), however, non-participation, loss to follow up and exclusion due to missing data constitute possible threats to the validity of the findings. Although non-participation in the UK census low, it is socially patterned such that more socio-economically disadvantaged households are less likely to participate [31]. It is also possible that more socio-economically disadvantaged ONSLS members were lost to follow-up between the 1991 and 2001 censuses. The children with missing data excluded from this analysis were more socially disadvantaged. Thus, this group was under-represented in the study sample and it is likely that our findings underestimate the impact of socio-economic disadvantage on the onset of disabling chronic conditions in childhood. The heritability of some conditions, particularly neuropsychiatric conditions, when combined with their association with socio-economic disadvanatage, may contribute to the observed social gradient.

Ten yearly censuses also artificially fix in time the measurement of the outcome when in reality, the development and identification of conditions and impairments is often a dynamic s process over the child’s lifecourse [32]. Furthermore, as with secondary analysis of any established dataset, there was no control over the data items collected. As a result, information on type and severity of disabling chronic conditions was not available and a limited set of potential confounding factors were available for study, leaving the possibility of residual confounding to account for observed differences.

## Conclusion

Disability is not simply the outcome of a particular impairment or health condition but rather a ‘dynamic interaction between health conditions and contextual factors, both personal and environmental’ [1]. Heritable and socio-economic factors come together in complex ways to increase a child’s risk of developing a chronic disabling condition [33]. While our findings add to the evidence base that exposure to socio-economic disadvantage in early childhood matters, further studies are needed to establish how long the exposure needs to last and to clarify pathways.There are at least three possible pathways. Children in socially disadvantaged households may be exposed to more social and environmental risk factors in the prenatal and early childhood periods that result in the later onset of activity limiting conditions [34]. Alternatively, they may have different experiences, that may increase the likelihood of impairments or conditions. For example, low household resources have been shown to affect parental capacity for supportive, stimulating and consistent parenting, which can be associated with poorer mental health, intellectual development and behavioural problems [35]. Reduced access, uptake or quality of services is a third possible pathway for socio-economically disadvantaged children in some countries [8], although in countries like the UK, that have universal access to free health care and education, and access to certain other services, this explanation is unlikely to account for the pattern reported in this paper. These pathways are not mutually exclusive. Elucidating how these contribute to the prevalence and manifestation of different disabling conditions is important in order to further refine preventative and supportive strategies to improve children’s lives [16].

Disabling chronic illness has a wide range of impacts on children and their families. While many disabled adults and children lead fulfilling lives and go on to make important social and economic contributions to society, many would accept that reducing the incidence of preventable disabling chronic conditions is desirable. This requires a multi-dimensional strategy that addresses both the factors that increase the incidence of health conditions and impairments leading to disability as well as social and environmental barriers that reduce social participation.^1^ Our study confirms the findings from other studies of child health, that targeting preventative efforts to reduce socio-economic disadvantage in early childhood is likely to be an important public health strategy to reduce health inequalities in later childhood and early adulthood. Although more research is needed, the evidence currently available tells us that developing and implementing policies that result in more favourable social and economic living conditions in early childhood are important [36]. Our findings indicate that the level of exposure to socio-economic disadvantage matters. Reducing poverty among households with young children is likely to remain key to achieving this. Many high income countries have unacceptably high child poverty rates. For example, in the UK in 2010/11, 27% of all children lived in low-income households [37], with the number of children experiencing material deprivation forecast to increase between 2010 and 2015 [38]. When many countries globally are experiencing economic crises and implementing austerity measures to reduce national debts, families with children are particularly vulnerable. Without policies and programes to tackle socio-economic disadvantage, the prevalence of disabling chronic conditions in childhood may increase, resulting in further demands on health, education and social services and putting the World Health Organisation’s aim of achieving health equity within a generation beyond the reach of many countries [39].

## Abbreviations

CI: Confidence interval; ONSLS: Office for National Statistics Longitudinal Study; OR: Odds ratio; SES: Socio-economic status; SDI: Socio-economic disadvantage index; UK: United Kingdom.

## Competing interests

The study was funded by a grant from the Economic and Social Research Council (ESRC), a UK independent, non-governmental public body. The funder did not have any role in the study design, analysis, interpretation or dissemination of research findings. The authors declare that they have no competing interests.

## Authors’ contributions

All authors contributed to the conceptualisation and design of study. CB and NS nterpreted the data. CB drafted and revised the manuscript. NS interpreted the secondary data analysis, and contributed to drafting the manuscript. JR contributed to drafting the manuscript. All authors read and approved the final manuscript.

## Pre-publication history

The pre-publication history for this paper can be accessed here:

http://www.biomedcentral.com/1471-2431/13/101/prepub
